# Use of thermal imaging to collect fresh faeces for non-invasive evaluation of stress levels in the European hare (*Lepus europaeus*)

**DOI:** 10.3389/fvets.2025.1682443

**Published:** 2025-11-20

**Authors:** Vlastimil Skoták, Jan Cukor, Marek Sedláček, Richard Ševčík, Rostislav Linda, Kateřina Brynychová, Matěj Kostka, Sabine Macho-Maschler, Jan Hušek, Rupert Palme

**Affiliations:** 1 Forestry and Game Management Research Institute, Jíloviště, Czechia; 2 Faculty of Forestry and Wood Technology, Mendel University in Brno, Brno, Czechia; 3 Faculty of Forestry and Wood Sciences, Czech University of Life Sciences in Prague, Prague, Czechia; 4 Central European Institute of Technology, Masaryk University, Brno, Czechia; 5 Experimental Endocrinology, Department of Biological Sciences and Pathobiology, University of Veterinary Medicine, Vienna, Austria; 6 Department of Zoology, National Museum of the Czech Republic, Prague, Czechia

**Keywords:** animal welfare, biodiversity conservation, cortisol, intrapopulation stress, non-invasive monitoring, wildlife monitoring

## Abstract

Assessing long-term stress in wild animal populations is extremely complicated and, in some species, practically impossible due to the complexity of sampling. Here, we tested and verified a unique non-invasive method for collecting fresh faecal samples located using thermal imaging cameras from a model species, the European hare. Subsequent analysis of faecal glucocorticoid metabolites (fGCMs) allows for the determination of stress levels without capture of individuals in hare populations. fGCM values ranged from 1.8 to 65.8 ng/g. Whilst the average value across locations in winter was 15.6 ng/g ± 9.9 SD, values in spring (April/March) were significantly higher (18.2 ng/g ± 11.2 SD; *p* < 0.05). These higher values coincided with the peak in European hare reproduction in spring. Significantly higher values were also confirmed in urban environments, indicating increased stress levels compared to natural environments, despite hares appearing to have adapted to the urban landscape. Higher values were also found in structurally poorer landscapes than Austrian rich agroecosystems. Having proved the usefulness of thermal imaging cameras for collecting large numbers of fresh droppings for the non-invasive evaluation of stress in wild hare sub-populations, we suggest the method could be applied to other species where capture and handling exert stress, injury or mortality.

## Introduction

Whilst the population density of wild animal species is usually directly related to the quality of the environment in which they live, it can be difficult to assess the direct impact of environmental quality on population density as it is influenced by many factors (1). One method that has been used to assess environmental suitability and population success, particularly in endangered species, is to compare stress levels between individuals (ind.), populations or sub-populations, particularly as regards the effects of long-term stress caused by declines in environmental quality (2, 3). Endocrinological studies that monitor stress hormone concentrations in blood as an indicator of physiological stress are common (4) and are used for a wide range of wild and domestic animal species and for assessing the welfare of animals kept in captivity. However, the method tends to be far more successful in the case of domestic animals, which are easier to handle.

Most methods today analyse stress hormones such as cortisol and corticosterone, steroid hormones produced by the adrenal cortex and derived from cholesterol (5). These glucocorticoids (GCs) directly affect sugar metabolism and thus play an important role in controlling the metabolism of almost all tissues in the body (6). The dominating glucocorticoid (cortisol or corticosterone) differs between species, where most mammals including the European hare (*Lepus europaeus*) have cortisol (7).

A wide range of sample types may be used to monitor GC levels, these typically include blood, saliva, excrement, milk, fur, feathers and eggs (8, 9). Unfortunately, the sampling process itself can cause considerable stress to wild animals as it usually involves capturing and handling. Furthermore, blood sampling can cause acute stress, which will then be reflected in the levels of stress hormones in the blood, giving a false indication of environmental stress (10). In such cases, it would be more appropriate to use a non-invasive sampling method to determine GC and their metabolites (11). However, a major limitation of non-invasive sampling is the difficulty of reliably identifying freshly deposited faeces. As shown by Lafferty et al. (12), exposure to environmental conditions can rapidly alter glucocorticoid metabolite concentrations, which highlights the importance of collecting only fresh samples to ensure reliable results. Collection of an animal’s faeces offers a particular advantage in that they are easy to collect. However, faecal (and urine) samples can be difficult to (a) identify to species level, and (b) link to a specific individual. Furthermore, circulating hormone levels in faecal samples are integrated over time and thus are representative of cumulative hormone secretion (9). Consequently, most modern studies now monitor faecal GC metabolite (fGCM) concentrations when using faecal (or urine) samples as a non-invasive method to evaluate stress in animal welfare assessments (13–16).

Stress levels will be affected by several factors, including environmental issues, which can have a significant impact on reproduction and population dynamics, especially in endangered species (17). Over the last century landscapes have been profoundly affected by human management worldwide, especially those associated with agroecosystems (18), which require a constant supply of energy in the form of fertilisers, pesticides and other agrotechnical interventions (19). These, often huge, agricultural operations have had a profound impact on wild animal populations, often representing the main limiting factor in present population dynamics (20, 21). Animal populations mainly affected by such operations include ground-nesting field birds, such as the grey partridge (*Perdix perdix*), the northern lapwing (*Vanellus vanellus*) and corncrake (*Crex crex*) (22), and mammals such as the European hare, whose population dynamics show a clear negative reaction to changes in the agricultural landscape (23). Though a relatively small species, the hare has relatively high environmental demands and, consequently, has been classified as a bioindicator species (24, 25). Just 60 years ago, the hare was a very common species in Central Europe (26), as documented by hunting catches of more than 1 million individuals per year in the Czech Republic alone. In recent decades, agricultural changes (e.g., a shift to large ‘prairie’ field systems and crop homogenisation) have had a demonstrable negative impact on hares, with numbers declining throughout Central Europe, even to the point of virtual extinction in some area (27–29). Despite this, there is a lack of information regarding the impact of environmental stress on European hare (sub-) populations, most such studies having focused on stress monitoring in snowshoe hares (*Lepus americanus*) (30–33) or mountain hares (*Lepus timidus*) (34, 35). In all studies, cortisol (or its faecal metabolites) has been monitored as the prime stress indicator in hares (36–40).

The aim of this study was to (i) test and verify a new method for identifying fresh faeces (infra-red monitoring), which can then be used to assess European hare stress levels based on analysis of fGCM concentrations (i.e., metabolites of cortisol); (ii) to compare stress levels in sub-populations from different agricultural landscapes with differing hare densities; (iii) to compare stress levels in hares from agricultural environments with those from urban environments, and (iv) to compare stress levels during the period of food scarcity (December/January) and the breeding season (February/March). We hypothesise that fGCM concentrations will decrease as landscape suitability (i.e., heterogeneity) increases.

## Materials and methods

### Study area

**Figure 1:**
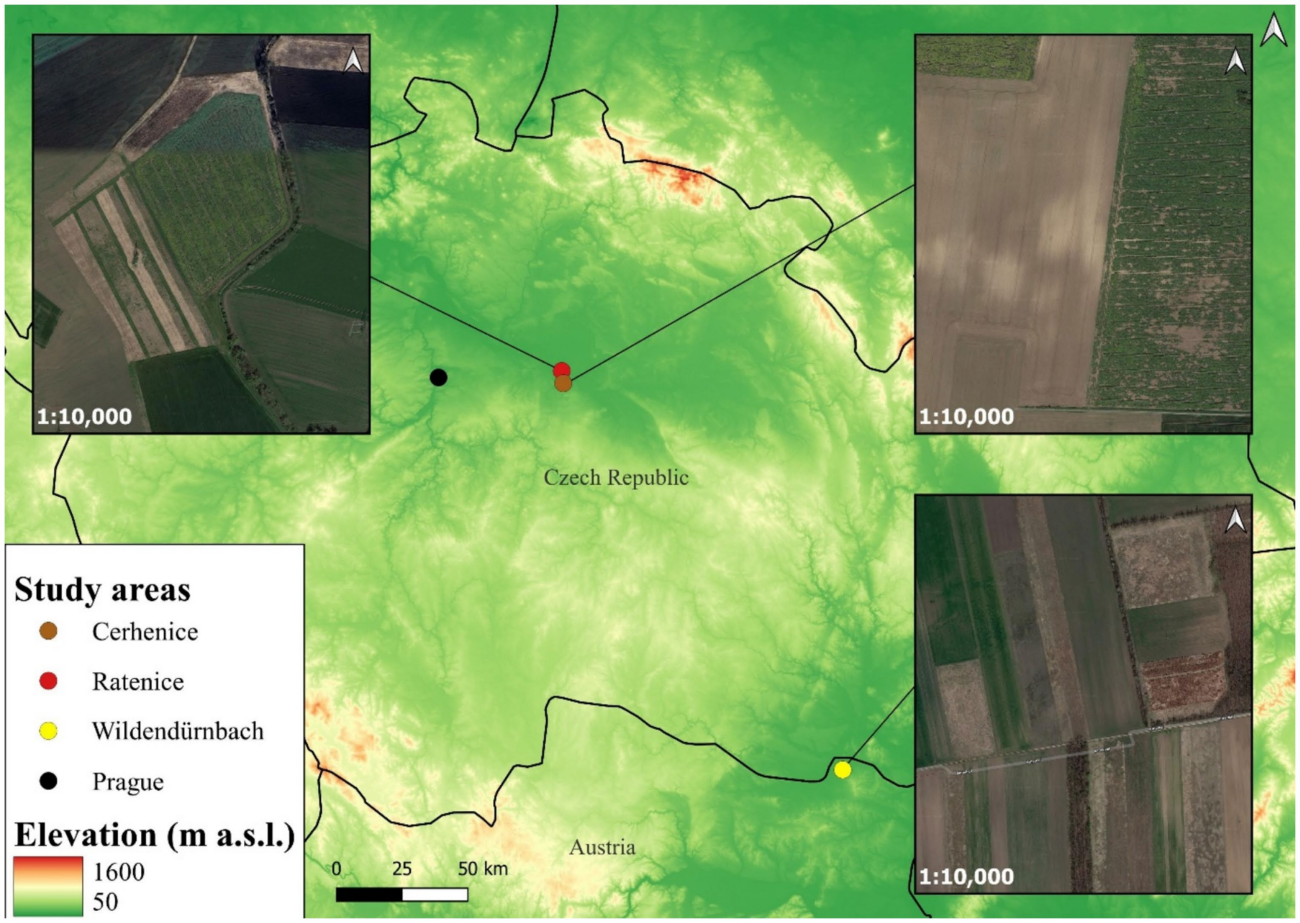
Location and landscape structure of the study areas.

Samples were obtained from two agricultural landscapes and one urbanised area in the Czech Republic and an additional agricultural site in Austria (Figure 1). The first agricultural site, located close to the village of Cerhenice (50.060278 N, 15.066389E; 240 m a.s.l.; Czech Republic), has a relatively homogeneous landscape and is characterised by conventional modern farming practices. In 2023, the average field size in this area was 13.13 ± 13.51 SD ha. The proportion of arable land is relatively high at 87.15%, with other non-agricultural landscape elements, such as wildflower strips (0.73%), almost absent (41). The second agricultural site, situated 1.1 km from the first site near the village of Ratenice (50.098611 N, 15.058750E; 188 m a.s.l.; Czech Republic), is managed using extensive farming practices and is characterised by a more heterogeneous landscape. The average field block size is smaller (4.64 ± 5.03 SD ha), the proportion of arable land reduced (83.01%) and the proportion of features such as wildflower strips higher (4.68%). The third agricultural site, located near Wildendürnbach (48.779167 N, 16.493611E; 193 m a.s.l.; Lower Austria), lies in a highly heterogeneous landscape with significantly smaller strip field blocks (1.78 ± 1.70 SD ha) with higher edge density, a lower proportion of arable land (64.83%) and a higher proportion of wildflower strips (5.93%) and other non-cropped elements (41). The final urbanised site is situated in the city of Prague (50.087500 N, 14.421389E; 192 m a.s.l.; Czech Republic), a large Central European city with a population of approximately 1,350,000 people.

Note that, whilst fGCM concentrations were determined for the urban location, they were not compared with those from the other sites due to the clear difference in environment type and the absence of current data on hare population density in such urban environments. Previous monitoring efforts have recorded average adult hare spring population densities in more heterogeneous localities (Ratenice, Wildendürnbach) are significantly higher (mean density ± SD = 117 ± 68 and 114 ± 49 ind./100 ha, respectively) than population density (30 ± 24 ind./100 ha) in more homogeneous locality (Cerhenice) (41).

European hare pellet samples were obtained during night patrols (i.e., the natural activity period of the species, between 21:00 and 02:00 during two consecutive nights at each site) at known activity centres with the use of Merger LRF XP50 thermal imaging binoculars (640 × 480 pixel microbolometer, NETD < 25 mK, F50/1.0 lens, 2.5–20 × magnification; Pulsar, Lithuania), which enabled real-time detection of both individuals and fresh pellets based on their heat signature, which made them clearly distinguishable from older pellets. The pellets were usually obtained from the hare’s last resting place as defecation inevitably occurred immediately after the individual was disturbed by the approach of a researcher. This method allowed for the collection of fresh droppings only, thereby minimising the potential risk of steroid degradation due to weather exposure. If multiple faecal pellets were produced by the same individual, all pellets were collected, thoroughly homogenized, and treated as one composite sample representing that individual (weight of the sample is specified below). Upon collection, the samples were immediately placed in test tubes, frozen at −20 °C in a field freezer and then stored at −20 °C until laboratory analysis.

### Sample analysis

For quantification of fGCMs, the samples were first dried at 75 °C for 5 h and then mechanically homogenised. For extraction, 0.20 g (± 0.005 g) of dry matter was mixed with 4.0 mL of 100% methanol and 1.0 mL of distilled water. The samples were then placed on a shaker for 30 min and subsequently centrifuged at 2500 g for 10 min, after which the supernatant was diluted at a 1:10 ratio with test buffer (13). Determination of fGCMs was performed using a group-specific 11-oxoaetiocholanolone enzyme immunoassay, which detects 11,17-dioxoandrostanes, a group of cortisol metaolites (13, 36, 40). For statistical evaluation purposes, samples with concentrations below the detection limit were set to 1.8 ng/g.

### Statistical analysis

For each location, the relationship between hare abundance (ind./100 ha) and (a) mean size of land patch, and (b) fGCM concentration across both study seasons (December/January and February/March), were assessed using the non-parametric Kruskal-Wallis test (assumptions of ANOVA not met), with subsequent multiple comparisons. Likewise, as assumptions of sample heterogeneity were not met in any sample, fGCM concentrations between study seasons for each location separately and study seasons across all locations were compared using the Wilcoxon rank-sum test, with results presented as boxplots. The correlation between acreage of particular field blocks and hare abundance was assessed using Pearson correlation coefficient and respective statistical test (test for correlation between paired samples).

All statistical procedures were performed using R software (42), with plots created using the R package “ggplot2” (43). An alpha level of 0.05 was used for all statistical tests.

## Results

**Figure 2:**
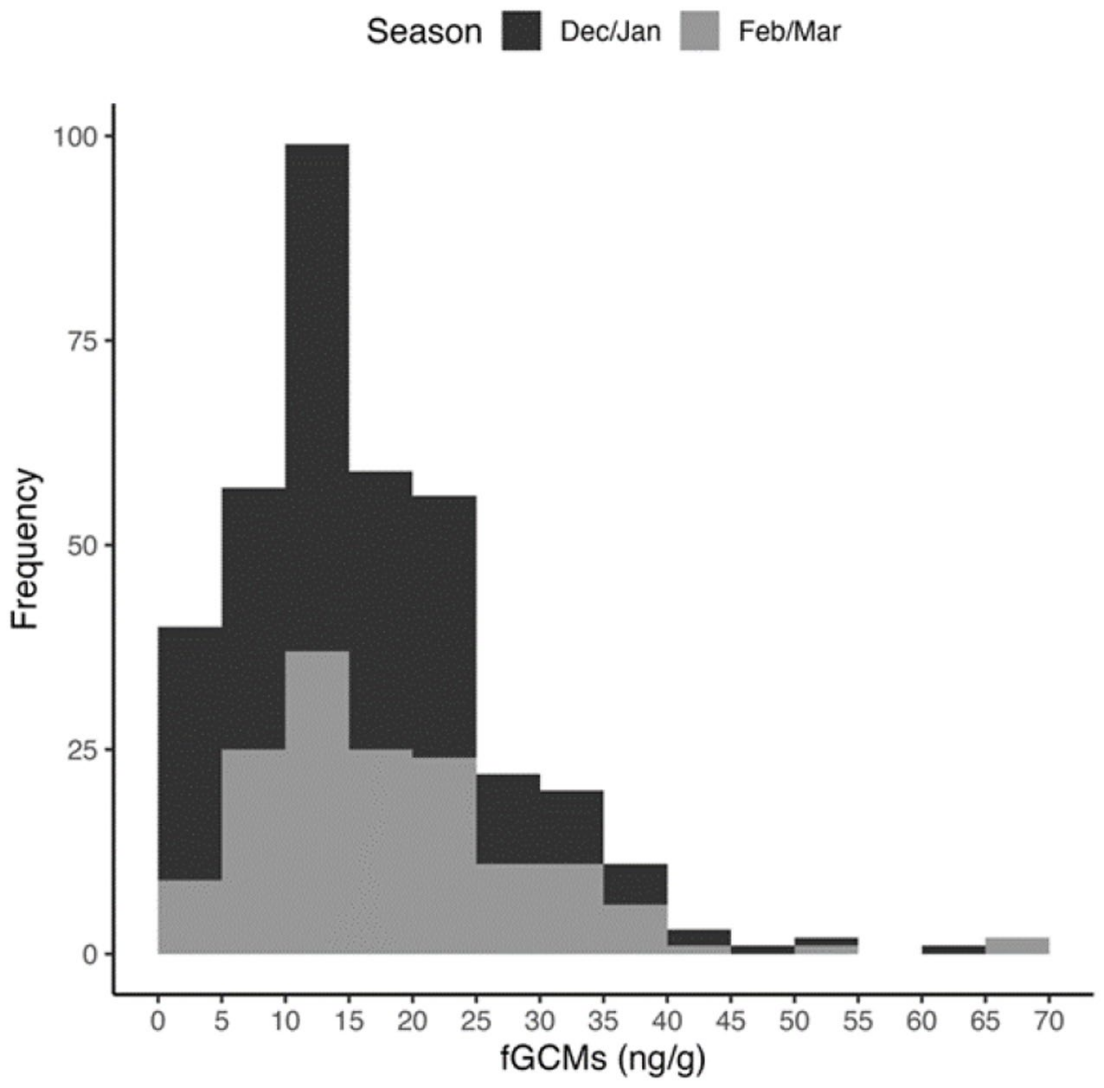
Histogram of fGCM concentrations detected across all locations.

**Figure 3:**
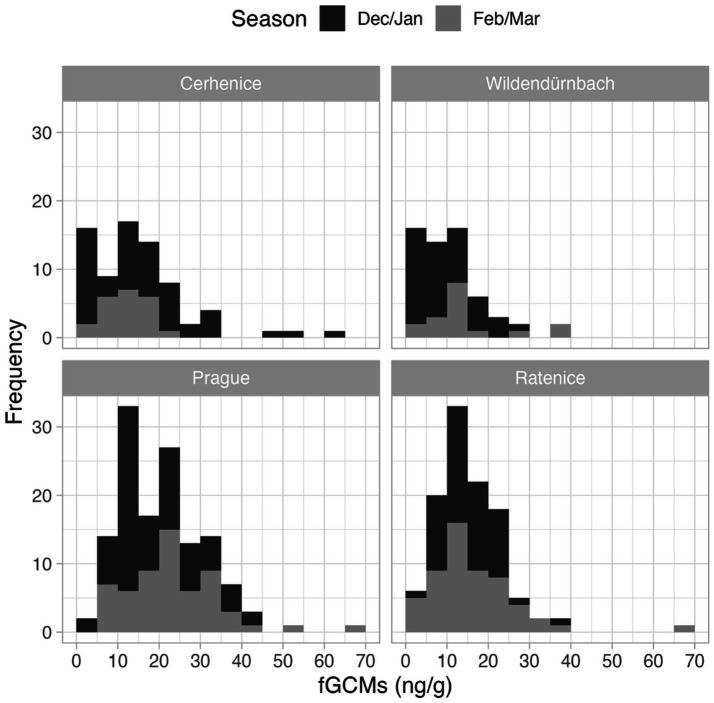
Histograms of fGCM concentrations detected at each location.

A total of 373 faecal samples were analysed, comprising 73 from Cerhenice, 132 from Prague, 59 from Wildendürnbach and 109 from Ratenice. Concentrations of fGCM ranged from 1.8 to 65.8 ng/g (average = 16.68 ng/g), though high values were relatively rare (Figure 2). Whilst values below the minimum detection limit of 1.8 ng/g were recorded at all locations except Prague, Prague had the maximum fGCM concentration of 65.8 ng/g recorded in the Czech Republic (spring), the maximum recorded in Wildendürnbach was 37.6 ng/g (Figure 3).

**Figure 4:**
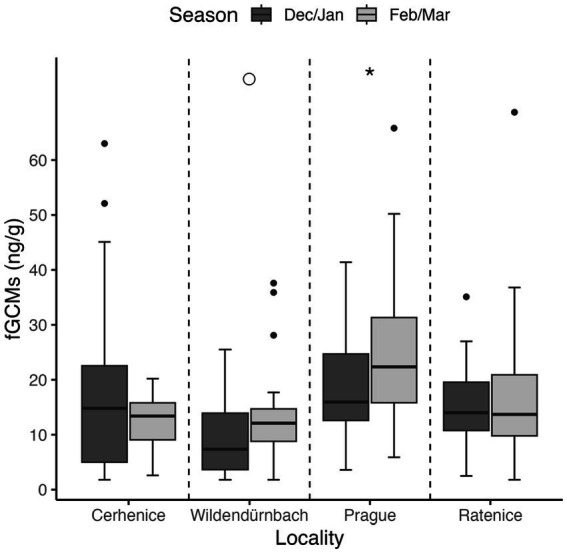
Boxplots of fGCM concentrations by location and sampling season. Symbols above the groups indicate statistical significance: * = *p* < 0.05, ○ = marginally significant (0.05 < *p* < 0.1), no symbol = no significant difference.

Lowest (mean ± SD) fGCM values were recorded in Wildendürnbach (10.8 ± 8.3 ng/g), whilst highest values were recorded in Prague (21.0 ± 10.5 ng/g), i.e., around twice those in Wildendürnbach. Comparable concentrations were recorded at the other Czech localities, with an average of 15.2 ± 11.5 ng/g at Cerhenice and 15.6 ± 8.8 ng/g at Ratenice. Overall (all sites combined; data not shown), fGCM concentrations were significantly higher in spring (winter: 15.6 ± 9.9 ng/g, spring: 18.27 ± 11.2 ng/g; W = 14,662, *p* < 0.05). However, no consistent seasonal trend was observed between localities, with concentrations comparable between seasons at Cerhenice and Ratenice and higher values in spring at Wildendürnbach and Prague (Figure 4), though the difference was only significant at Prague (*p* < 0.05).

**Figure 5:**
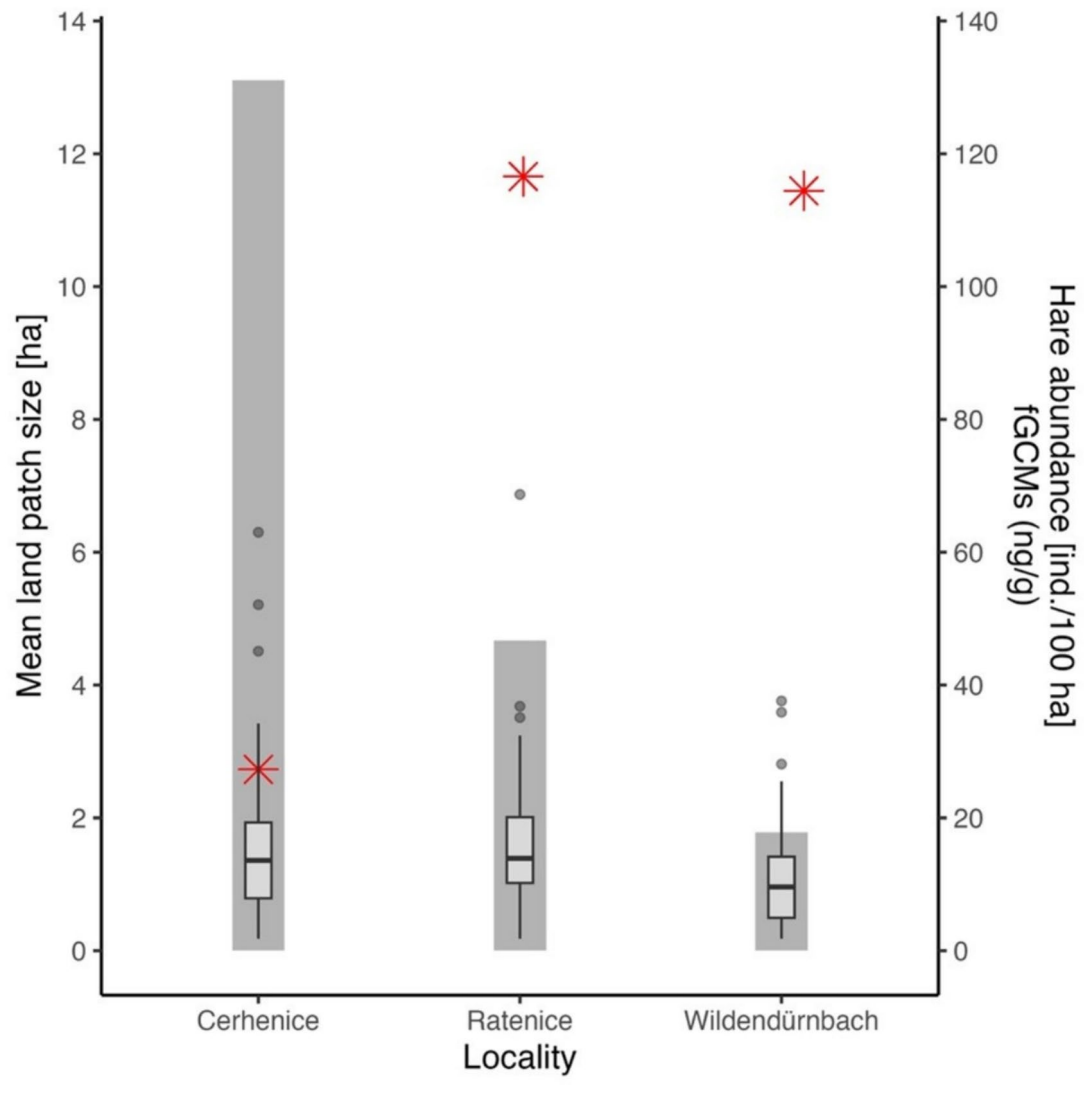
Average field block size (columns, primary axis on left), hare abundance (red stars, secondary axis on right) and fGCM concentration (boxes, secondary axis on right) at each of the rural localities.

A comparison of average field block size, fGCM concentration (across sampling dates) and hare abundance at the three rural sites (urban location excluded; Figure 5) indicated a high negative correlation between field block size and hare abundance (*r* = −0.975), though the result was not statistically significant due to the low number of sites assessed (*p* = 0.14). There was a highly significant difference in fGCM concentrations between sites (Kruskal-Wallis test, chi-squared = 15.499, df = 2, *p* < 0.001), with multiple comparisons showing values from Wildendürnbach to be significantly lower than those at Czech sites, which showed no significant difference.

## Discussion

Over the past 20 years, the determination of fGCMs has become an increasingly popular method for assessing stress in animals, because faeces collection is non-invasive and avoids additional stress due to capture and handling (8). However, faeces need to be collected fresh, because fGCMs were found to degrade easily (16), which is especially challenging in free ranging, wild animals. In this study, we tested a new method for ensuring the collection of fresh wild hare pellets, and used those to determine environmental suitability for hares by relating fGCM concentrations (as an indicator of stress) (35, 44) to the density of a given sub-population.

In the case of European hare, studies on captured hares have shown that the related acute stress poses a particularly serious risk as it can lead to trauma or mortality (45). Such influences can be minimised, if fGCMs are measured in samples collected without disturbing the animal. However, age of faecal samples can be crucial in ensuring accurate fGCM readings as levels may change over time due to degradation by bacterial enzymes (16, 43, 46–48). Time after defecation and environmental conditions have been found to exert a strong influence on measured fGCM levels (16, 43, 49). Therefore, the generally accepted advice is to collect fresh faeces (13). This may, however, be a serious problem in wildlife studies. Here we report for the first time the use of thermal imaging camera not only for detecting the animals, but also the freshly deposited faecal pellets. By using mobile freezer boxes, we were also able to freeze samples immediately on site, preventing any subsequent degradation (50).

Likewise, by sampling always at night with the thermal camera, a possible influence of a diurnal rhythm in GC secretion was avoided (8, 51). One limitation of the thermal camera method is that it is not possible to determine the sex of individuals due to a lack of sexual dimorphism in the case of European hare, and this may affect GC concentrations (8, 35, 50). This will not be an issue, however, if the method is applied to other wild species with more obvious sex dimorphism, easily observable with a thermal camera. For example, in some mammalian species such as red deer (*Cervus elaphus*), fallow deer (*Dama dama*), or mouflon (*Ovis musimon*), infrared thermography can reliably distinguish males by the presence of antlers or horns and by differences in body morphology.

In our study, which evaluated 373 faecal samples from wild European hares, fGCM levels ranged from 1.8 to 65.8 ng/g, with an average concentration of 16.7 ng/g. These values correspond with those from a comparable study by Cybulska et al. (36), who monitored fGCM concentrations in European hares in an agricultural landscape in Wildendürnbach during the grain harvest. Using 591 faecal samples, they recorded concentrations ranging between 5 and 65.6 ng/g. In another study on captive European hares, Teskey-Gerstl (40) recorded baseline concentrations ranging from 43 to 274 nmol/kg, equivalent to 13.0 to 82.9 ng/g. By applying a stress stimulus (repeated disturbance of rest), they were able to induce metabolite levels up to five times higher than original baseline values, with maximum concentrations reached within 1–2 days of stress stimulus, after which levels gradually returned to baseline. However, that these values refer to cage-bred hares only, where levels of stress can be induced or influenced and subsequently monitored relatively easily (51). To determine levels of stress associated with artificial breeding. Janicki et al. (52) monitored fGCM levels in pellets of European hares kept as pairs in cages. They recorded average values of around 201 ng/g in May and 180 ng/g in November, with maxima exceeding 300 ng/g. Such concentrations were significantly higher than those from wild individuals. The authors attributing this to constant mild stress caused by the unnatural cohabitation of males and females in a confined space. Consequently, stress hormone concentrations from captive studies should not be compared directly with wild animal studies or those using other types of samples.

In the wild, stress levels are assessed to evaluate intraspecific stress or the welfare status of a given species in relation to environmental quality. In our study, we recorded average fGCM concentrations of 10.8 ng/g at a highly heterogenous site in Wildendürnbach, and a significantly higher value of 21.0 ng/g at the homogenous urban site in Prague, i.e., around double the Austrian average. Four outliers (> 50 ng/g) are likely explained by possible trauma, possibly resulting from attempted predation. In a study by Boudreau et al. (30), however, no increase in fGCM levels (against a control) was recorded in hares exposed to predation risk. Further analysis was based on a comparison of the rural sites only as it was not possible to determine hare population density accurately in Prague due to the highly urbanised nature of the city. Consequently, we base our findings solely on comparisons of the Wildendürnbach locality (avg. 10.8 ng/g), Cerhenice (avg. 15.15 ng/g) and Ratenice (avg. 15.63 ng/g). Whilst the population density of the European hare differed significantly (*p* < 0.001) between the Czech homogenous landscape (Cerhenice) and two heterogenous landscapes in the Czech Republic (Ratenice) and Austria (Wildendürbach) (41), the differences in metabolite levels were not significant. Thus, our results do not indicate that stress levels in hares are lower at localities with lower population density. Instead, stress level changes are more likely to be caused by unsuitable agricultural landscape structure (23, 44, 53). This is particularly clear when comparing urban environments like Prague with highly structured rural landscapes, which represent ideal environments for hares (26). Surprisingly, previous studies have recorded a relatively high hare population density in urban environments (ca. 8 ind./100 ha, including building areas) (54), with the hare showing considerable adaptability to human presence in urban environments, their flight distance being less than half of that in agricultural landscapes (55). Nevertheless, evaluation of stress hormones shows that, despite their apparent tolerance, individuals living in urban environments are likely to suffer from long-term, low-level stress. Our results also showed a notable effect of field block size on stress levels, with a trend of fGCM concentrations increasing as field block area increased. However, this relationship was only significant for the Wildendürnbach site (*p* < 0.05). This suggests that large land blocks, often associated with intensive agricultural management and low landscape diversity, increase stress levels in hares, probably due to the relative lack of shelter options and the higher risk of disturbance/predation.

In the present study, slight seasonal differences in fGCM levels were observed, with higher concentrations in February/March compared to December/January, most likely related to hares mating in spring. Whilst male hares are fertile for most of the year (except October and November), females are limited to an extended reproductive season stretching from January to August, peaking in March (56). At this point, females become highly receptive, prompting high levels of aggressive competitive activity, a phenomenon often referred to as “March madness” [e.g., see Lincoln (56)]. During this month, when 100% of adult females usually become pregnant, the high levels of competition are likely to result in both physical exhaustion and associated stress, especially in females. This is the most likely explanation for the higher stress levels observed in sub-populations with higher population densities observed in this study, where competition for females is likely to be more intense. As such, the results partially confirm our hypothesis that higher density populations show higher levels of stress, albeit seasonally. However, we compared only spring data with that from December/November, when hares may already be entering their breeding season. Increased fGCM concentrations in spring may partly reflect elevated physiological stress in pregnant females, potentially linked to adaptive maternal effects as shown in other mammal species (57). Thus, we suggest that further sampling may be appropriate in October/November, allowing a better separation of mating-related stress and environmental stress levels.

## Conclusion

The results of this study confirm high quality thermal imaging binoculars as a valuable tool for identifying a model animal species, the European hare, at night in the wild, allowing for the non-invasive collection of fresh faecal pellets for determining fGCM, without the need for trapping or handling. As such, we highly recommend its use for collecting fresh faeces in other sensitive or endangered species where disturbance or handling could adulterate subsequent stress levels.

The fGCM values recorded indicated a relatively balanced level of adrenocortical activity in open agricultural landscapes, but higher stress values in urban environments. Whilst the hare shows a high level of adaptability in urban areas, they are clearly not comfortable in such environments. Further, we recorded lower stress levels in hares from a heterogenous extensively farmed landscape (Wildendürnbach) compared with more conventionally managed agricultural landscapes (Czech Republic), confirming that larger, more intensively managed field blocks represent less favourable wildlife environments. Finally, our study confirmed increased stress levels in hares during the spring reproductive period, though we were unable to separate data by sex and our comparative autumn data did overlap slightly with potential reproduction.

Overall, our study confirms the suitability of thermal imaging as a non-invasive monitoring tool and that fGCM assessment in faecal matter obtained in this way can be a sensitive indicator of physiological stress when monitoring the impact of landscape structure and agricultural management on wildlife species. Longer-term monitoring at more locations and at different times of year could be beneficial in gaining a deeper understanding of the relationships between stress physiology, environment and hare behaviour.

## Data Availability

The raw data supporting the conclusions of this article will be made available by the authors, without undue reservation.
